# A Link between Intrahepatic Cholestasis and Genetic Variations in Intracellular Trafficking Regulators

**DOI:** 10.3390/biology10020119

**Published:** 2021-02-04

**Authors:** Qinghong Li, Yue Sun, Sven C. D. van IJzendoorn

**Affiliations:** Department of Biomedical Sciences of Cells and Systems, Section Molecular Cell Biology, University of Groningen, University Medical Center Groningen, 9713 GZ Groningen, The Netherlands; q.li@umcg.nl (Q.L.); y.sun@umcg.nl (Y.S.)

**Keywords:** intrahepatic cholestasis, VPS33B, VIPAR, MYO5B, AP1S1, SCYL1, intracellular trafficking, hepatocyte, recycling endosome

## Abstract

**Simple Summary:**

Cholestasis refers to a medical condition in which the liver is not capable of secreting bile. The consequent accumulation of toxic bile components in the liver leads to liver failure. Cholestasis can be caused by mutations in genes that code for proteins involved in bile secretion. Recently mutations in other genes have been discovered in patients with cholestasis of unknown origin. Interestingly, many of these newly discovered genes code for proteins that regulate the intracellular distribution of other proteins, including those involved in bile secretion. This group of genes thus suggests the deregulated intracellular distribution of bile-secreting proteins as an important but still poorly understood mechanism that underlies cholestasis. To expedite a better understanding of this mechanism, we have reviewed these genes and their mutations and we discuss these in the context of cholestasis.

**Abstract:**

Intrahepatic cholestasis is characterized by the accumulation of compounds in the serum that are normally secreted by hepatocytes into the bile. Genes associated with familial intrahepatic cholestasis (FIC) include *ATP8B1* (FIC1), *ABCB11* (FIC2), *ABCB4* (FIC3), *TJP2* (FIC4), *NR1H4* (FIC5) and *MYO5B* (FIC6). With advanced genome sequencing methodologies, additional mutated genes are rapidly identified in patients presenting with idiopathic FIC. Notably, several of these genes, *VPS33B*, *VIPAS39*, *SCYL1*, and *AP1S1*, together with *MYO5B*, are functionally associated with recycling endosomes and/or the Golgi apparatus. These are components of a complex process that controls the sorting and trafficking of proteins, including those involved in bile secretion. These gene variants therefore suggest that defects in intracellular trafficking take a prominent place in FIC. Here we review these FIC-associated trafficking genes and their variants, their contribution to biliary transporter and canalicular protein trafficking, and, when perturbed, to cholestatic liver disease. Published variants for each of these genes have been summarized in table format, providing a convenient reference for those who work in the intrahepatic cholestasis field.

## 1. An Introduction to the Enterohepatic Circulation of Bile Acids and Familial Intrahepatic Cholestasis 

**Figure 1:**
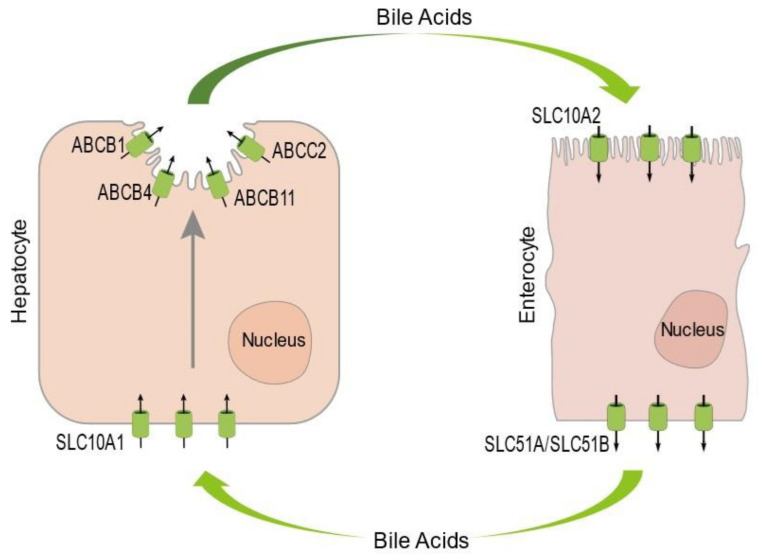
Cartoon illustrating the entero-hepatic circulation of bile acids, the polarized distribution of the canalicular proteins ABCB1, ABCB4, ABCB11 and ABCC2 and the basolateral protein SLC10A1 in hepatocytes, and the polarized distribution of the brush border protein SLC10A2 and the basolateral proteins SLC51A and -B in enterocytes. Arrows indicate direction of movement (see text for further explanation).

In the healthy liver, hepatocytes synthesize bile acids, and secrete these via ATP Binding Cassette (ABC) subfamily B member 11 (ABCB11; or bile salt export pump (BSEP)), ABC subfamily C member 2 (ABCC2; or multidrug-resistance-associated protein (MRP)2) and ABC subfamily B member 1 (ABCB1, or multidrug-resistance protein (MDR)1) at their apical plasma membrane domains into the bile canaliculi [1]. These bile canaliculi are sealed by tight junctions which prevent the leakage of the bile canalicular contents into the blood-filled sinusoids. The secreted bile acids are moved via intra- and extrahepatic bile ducts and the gallbladder to the duodenum, where they aid in fat absorption. In addition, bile acids function as signaling molecules with important functions in the regulation of lipid, glucose and energy metabolism, inflammation, drug metabolism and detoxification [2]. Approximately 95% of the bile acids are reabsorbed in the ileum via solute carrier (SLC)10A2 (or apical sodium bile acid transporter (ASBT)) [3]. The reabsorbed bile acids leave the ileal enterocytes at their basolateral side via SLC51A and SLC51B (or organic solute (OST)α/β) transporters [1]. The bile acids then travel via the portal vein back to the liver, where they are taken up from the sinusoids into the hepatocytes via SLC10A1 (or basolateral sodium taurocholate cotransporting polypeptide (NTCP)) transporter [4,5], thereby completing the enterohepatic circulation [6]. Importantly, the polarized distribution of apical and basolateral bile acid transporters in the hepatocytes and enterocytes thus is necessary for the enterohepatic circulation of bile acids [7] (Figure 1). 

The expression of the bile acid transporters and bile acid synthesis are co-regulated [8]. Bile acids entering the ileal enterocytes can bind a bile acid receptor also known as the Farnesoid X receptor (FXR), which is a nuclear transcription factor. Ileal FXR activation reduces the de novo synthesis of SLC10A2, thereby limiting ileal bile acid uptake from the gut lumen [9]. Ileal FXR activation additionally induces the synthesis and basolateral secretion of fibroblast growth factor-19, which, upon its arrival in the liver via the portal vein, signals to the hepatocytes to lower de novo bile acid synthesis. Bile acids also bind FXR in the hepatocytes [10], resulting in a reduction in the expression of SLC10A1 [11] and an increase in the expression of ABCB11 [12], which jointly favor a reduced bile acid load in the liver cells. 

Intrahepatic cholestasis refers to a hepatocellular disorder related to alterations in bile acid load, bile salt transport, membrane fluidity, cytoskeletal function and/or tight junctions, such that hepatocytes are unable to metabolize and excrete bile or prevent the leakage of the excreted bile from the bile canaliculi. This results in the accumulation of bile constituents such as bile acids, bilirubin, copper and metabolized drugs in the serum [13]. Intrahepatic cholestasis, which often clinically presents with jaundice and pruritus, covers a broad clinical spectrum, from benign recurrent episodes of mild cholestasis to severe and progressive cholestasis leading to cirrhosis and liver failure. Intrahepatic cholestasis can be acquired or inherited (familial). Here we focus on familial intrahepatic cholestasis.

A common form of familial intrahepatic cholestasis (FIC) is progressive FIC (PFIC)-type 2 (PFIC2) [14]. PFIC2 is caused by variants in the *ABCB11* gene, which encodes the canalicular bile salt transporter ABCB11 [15]. *ABCB11* variants may impair the folding and subsequent trafficking of newly synthesized ABCB11 to the canalicular domain of the hepatocytes, or impair the bile acid transport activity of the ABCB11 protein at the canalicular domain [16,17]. Other forms of PFIC include PFIC1 caused by variants in the *ATP8B1* gene [18], PFIC3 caused by variants in the *ABCB4* gene [19,20], PFIC4 caused by variants in the *TJP2* gene [21,22], PFIC5 caused by variants in the *NR1H4* gene [23] and PFIC6 caused by variants in the *MYO5B* gene [24,25,26]. *TJP2* encodes the tight junction-associated protein zona occludens (ZO)2, and its loss of function is believed to compromise the tight junction function leading to the paracellular leakage of bile [21,22]. The importance of tight junctions in securing normal bile flow is supported by the recent identification of pathogenic variants in *USP53*, encoding a member of the deubiquitinating enzyme family that colocalizes and interacts with ZO2 at tight junctions in epithelial cells [27], in a patient with idiopathic FIC [28]. *NR1H4* encodes FXR, and pathogenic *NR1H4* variants are associated with the loss of ABCB11 expression [23]. *MYO5B* encodes myosin Vb, a molecular motor implicated in protein trafficking [26], as further discussed below. *ABCB4* encodes ABCB4 (or multidrug-resistance protein MDR3), which localizes at the canalicular membrane and is essential for the secretion of phospholipids (phosphatidylcholine) from hepatocytes into bile. It is believed that reduced ABCB4-mediated phospholipid secretion increases the toxicity of secreted bile, which in turn damages the hepatocytes. The absence of phosphatidylcholine secretion due to ABCB4 defects also leads to the formation of cholesterol crystals/gallstones, which can lead to the obstruction of bile ducts. *ATP8B1* encodes the ATPase Class 1 type 8B member 1 (ATP8B1, or FIC1), which localizes at the canalicular membrane and is believed to be an aminophospholipid flippase [29]. The mechanisms via which ATP8B1 mutants contribute to cholestasis are not clear. In addition to PFIC1 and -2, benign recurrent forms of intrahepatic cholestasis (termed BRIC) and intrahepatic cholestasis of pregnancy have also been associated with *ATP8B1* and *ABCB11* mutations. PFIC3 can be differentiated from the other PFICs by the detection of elevated serum levels of the liver enzyme gamma glutamyltransferase (GGT) which is indicative for liver damage. 

## 2. An increasing Number of FIC-Associated Genes Are Involved in the Regulation of Intracellular Protein Trafficking

Recently, other mutated genes have been identified in patients that presented with idiopathic FIC but tested negative for variants in *ABCB11*, *ABCB4*, *ATP8B1*, *TJP2*, *NR1H4* or *MYO5B*. These cases of idiopathic FIC can appear as parts of more complex syndromes. Interestingly, several of these FIC-associated genes, similar to *MYO5B*, are functionally associated with recycling endosomes and/or the Golgi apparatus, which are components of a complex process that controls the sorting and trafficking of plasma membrane proteins. These genes include *VPS33B* [30], *VIPAS39* [31], *AP1S1* [32] and *SCYL1* [33]. Together with the variants in *MYO5B* [24,25,26], the pathogenic variants in these genes suggest that defects in intracellular trafficking take a prominent place in FIC. 

Below, we first briefly summarize current knowledge on the intracellular trafficking of bile canalicular protein, in particular that of ABCB11. We then discuss the recently identified FIC-associated genes that are involved in the regulation of intracellular protein trafficking, their variants, their roles in traffic control and their potential contribution to cholestasis. Gene variants were retrieved from peer-reviewed articles by searching the PubMed database (https://www.ncbi.nlm.nih.gov/pubmed) using the following search string: [gene name] AND [cholestasis] (where [gene name] was either VPS33B, VIPAS39, MYO5B, AP1S1 or SCYL1). The variants are listed in Supplemental Table S1. 

### 2.1. Intracellular Trafficking of ABCB11 and Other Bile Canalicular Proteins

Pioneering work of the laboratories of Hubbard and Arias laid down the map of intracellular trafficking routes for bile canalicular proteins in hepatocytes (for an excellent review see Ref. [34]). 

Newly synthesized single membrane-spanning and glycosylphosphatidylinositol (GPI)-anchored canalicular proteins are transported from the *trans*-Golgi network first to the basolateral, sinusoidal plasma membrane domain. From here, canalicular proteins are endocytosed via different routes [35] and subsequently transported via a process called transcytosis to the apical, canalicular plasma membrane domain [36,37]. The transcytotic route in hepatocytes involves several compartments, among which is a common recycling endosome and a population of RAB11A-positive subapical recycling endosomes (ARE). Common recycling endosomes act as a second sorting site for non-canalicular proteins that were retrieved from the sinusoidal surface, while ARE appear dedicated to the apical delivery of canalicular proteins [38,39]. Several factors have been identified that regulate the transcytotic delivery of proteins to the canalicular membrane, including glycolipids and cholesterol [40,41], myelin and lymphocyte protein (MAL)2 [42], and the recycling endosome-associated small guanosine triphosphatases (GTPases) RAB3D [43] and RAB17 [44]. RAB17 was found to interact with the soluble N-ethylmaleimide-sensitive factor activating protein receptor (SNARE) syntaxin-2 [45], and GTP-bound, mono-SUMOylated RAB17 was shown to mediate the docking and fusion of transcytotic vesicles carrying single membrane-spanning and GPI-anchored proteins at the canalicular plasma membrane [44]. 

In contrast to single membrane-spanning and GPI-anchored proteins, newly synthesized polytopic canalicular proteins are transported from the Golgi apparatus to the canalicular domain via a route that does not involve the sinusoidal domain [46]. These proteins include ABCB1, the bilirubin transporter ABCC2, and ABCB11. In the biosynthetic route, canalicular transporters first travel through the endoplasmic reticulum, where they are glycosylated [47,48]. PFIC2-associated ABCB11 mutants that cannot be *N*-glycosylated are unstable and are degraded [48,49]. The modified ABCB11 then travels through the Golgi apparatus before being sorted at the trans-Golgi network for delivery to the canalicular domain. The trafficking of ABCB11 from the Golgi apparatus to the canalicular domain was shown to require p38 mitogen-activated protein kinase activity, as pharmacological inhibition of this kinase retained ABCB11 in the Golgi apparatus [50]. The trafficking of ABCB1 from the Golgi apparatus to the canalicular domain was shown to require protein kinase A and glucosylceramide [51]. 

In contrast to ABCB1 and ABCC2, newly synthesized ABCB11 en route from the trans-Golgi network to the canalicular plasma membrane is transiently sequestered in intracellular compartments [46]. Subcellular fractionation studies support that ABCB11 resided both at the canalicular plasma membrane and in intracellular compartments. Multiple intracellular ABCB11 pools may exist, and one of these was identified as RAB11A-positive ARE. These RAB11A-positive ARE typically localize just beneath the canalicular plasma membrane. Live-cell imaging revealed that ABCB11 constantly and rapidly exchanges between RAB11A-positive ARE and the canalicular plasma membrane [52]. This process appears to be tightly linked to the biogenesis of bile canalicular plasma membrane domains [53]. Whether RAB17 plays a role in the docking and fusion of transport vesicles carrying polytopic transporters at the canalicular plasma membrane is not known. 

Importantly, the mobilization of ABCB11 from sub-apically localized compartments to the canalicular plasma membrane could be stimulated when hepatocytes were exposed to taurocholate, or upon elevation of intracellular adenosine 3′,5′-cyclic monophosphate (cAMP) levels [54]. It was proposed that the regulated mobilization from RAB11A-positive ARE could serve to control the abundance of ABCB11 and other apical transporters at the canalicular plasma membrane, in this way tuning the required bile flow [54]. Mobilization from intracellular pools is likely balanced with the retrieval of proteins from the canalicular plasma membrane into the endosomal system, which includes RAB11A-positive ARE [55]. Conceivably, imbalances in the exchange rate between the canalicular plasma membrane and RAB11A-positive ARE in favor of the latter result in an unmet need for bile salt transporters at the canalicular plasma membrane. The resultant incapacity to secrete bile acids then leads to intrahepatic cholestasis [56]. 

In support of this, the exposure of hepatocytes to the estrogen metabolite estradiol-17beta-d-glucuronide (E17G) or to taurolithocholate induces acute cholestasis associated with the retrieval of canalicular ABCB11 and ABCC2 into RAB11A-positive ARE [57,58]. Following an E17G-induced cholestatic insult, ABCB11 and ABCC2 are internalized from the canalicular plasma membrane via clathrin-mediated endocytosis, which involves their interaction with the clathrin adaptor protein complex (AP)-2 and calcium-dependent protein kinase C signaling [59]. The endocytosed transporters are then either targeted for degradation in lysosomes or diverted to RAB11A-positive ARE. Additionally, the trafficking of these RAB11Aa-positive ARE to the canalicular plasma membrane is inhibited by E17G [58], thereby reinforcing the redistribution of canalicular ABCB11 to RAB11A-positive ARE. Interestingly, the E17G-induced sequestering of ABCB11 in RAB11A-positive ARE could be reversed by the treatment of the cells with glucagon [57]. Tauroursodeoxycholate (TUDC), which is a frequently used drug for cholestatic liver disease in humans, was shown to stimulate the mobilization of ABCB11 from sub-canalicular compartments to the canalicular plasma membrane [60]. Additionally, the cAMP- and TUDC-induced translocation of ABCC2 to the plasma membrane was reported to involve RAB11A [61].

It thus appears that the intracellular trafficking of ABCB11 and other canalicular proteins, and in particular the involvement herein of RAB11A-positive ARE, plays a pivotal role in the regulation of canalicular function and, when perturbed, in cholestatic liver disease. Because these trafficking pathways may be druggable targets for the treatment of intrahepatic cholestasis, a detailed understanding of the precise molecular machineries that control these is warranted. The identification and study of FIC-associated “trafficking” genes offers a (patho-)physiologically relevant starting point. 

### 2.2. VPS33B and VIPAS39

**Figure 2:**
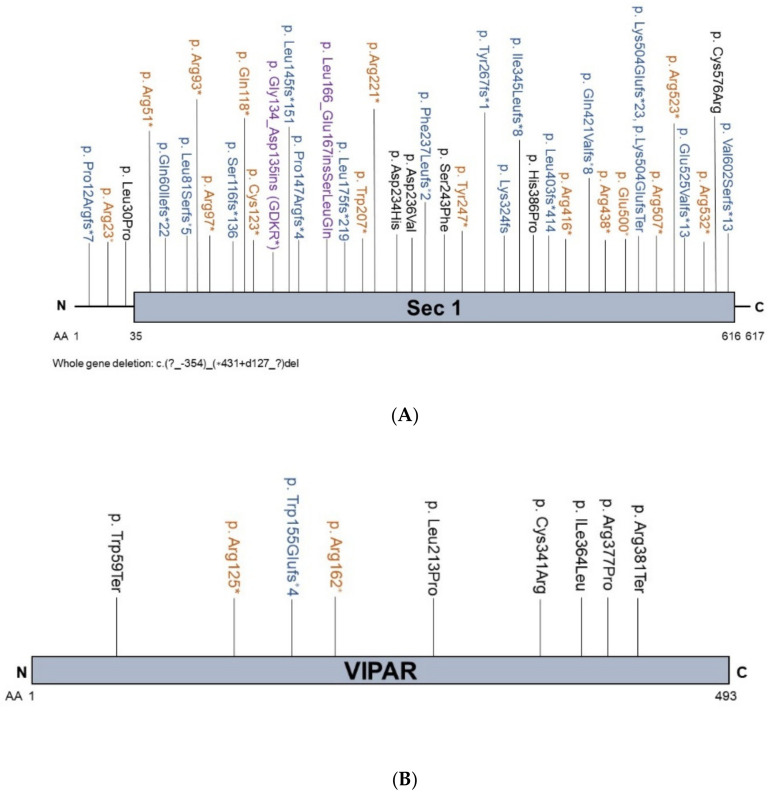
Overview of VPS33B and VIPAR protein mutations associated with cholestasis. (**A**): Cholestasis-associated mutations in the VPS33B protein and its domains. The SEC1 domain of VPS33B is shown. (**B**): Cholestasis-associated VIPAR protein mutations. N: aminoterminus; C: carboxylterminus. AA: amino acid; *: premature termination.

*VPS33B* [30] or *VIPAS39* [31] variants (Supplemental Table S1, Figure 2A,B, respectively) were identified in patients with a rare multisystem disorder called arthrogryposis-renal dysfunction-cholestasis (ARC) syndrome. Approximately 75% and 25% of ARC patients have predicted pathogenic variants in *VPS33B* or *VIPAS39*, respectively. Patients with ARC display heterogeneous clinical presentations, but commonly present with neonatal cholestasis with low GGT activity [62]. One ARC patient was reported with high GGT cholestasis [63]. Recently, *VPS33B* variants were identified in patients that presented cholestasis but no other clinical features of classic ARC [64], underscoring a strong association between *VPS33B* and cholestasis.

**Figure 3:**
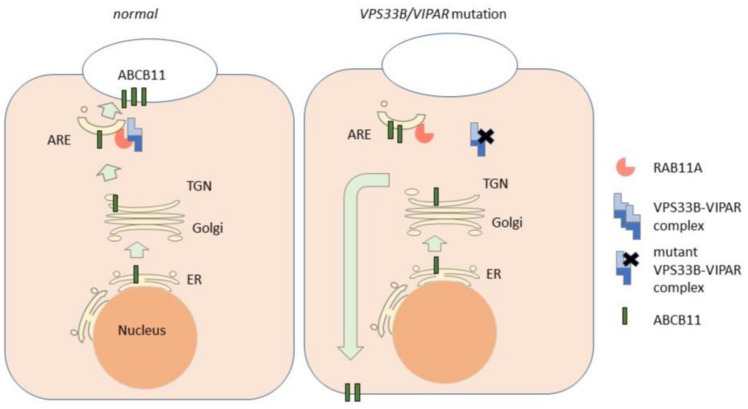
Cartoon depicting the role of the VPS33B/VIPAR complex and its mutations in the trafficking of ABCB11. In the normal situation (left), newly synthesized ABCB11 traffics via the endoplasmic reticulum (ER), the Golgi apparatus and the trans-Golgi network (TGN) to the apical recycling endosomes (ARE). From the ARE, ABCB11 is trafficked to the bile canalicular surface via a mechanism that involves the ARE-associated RAB11A and its interaction with the VPS33B/VIPAR complex. In the absence of mutated forms of VPS33B or VIPAR, the RAB11A–VPS33B/VIPAR complex cannot form, resulting in the impaired trafficking of ABCB11 to the canalicular surface and the mis-sorting of ABCB11 to the basolateral surface.

*VPS33B* is located at region 2, band 6, sub-band 1 on the long q-arm of chromosome 15 (15q26.1), and encodes vacuolar protein sorting-associated protein 33B (VPS33B). VPS33B contains a mammalian uncoordinated-18 (munc18)/secretion mutant-1 (sec-1) domain which is shared with other proteins that regulate membrane fusion events during vesicular trafficking. *VIPAS39* is located on chromosome 14q24.3 and encodes the VPS33B-interacting protein and apical-basolateral polarity regulator (VIPAR). VPS33B and VIPAR proteins, when together in a complex, localize in cells to the endosomal system, in particular to ARE, as marked by the small GTPase RAB11A (Figure 3). RAB11A-positive ARE are positioned close to the apical bile canalicular domain of hepatocytes, and are implicated in the hepatocyte polarity and bile canalicular functions [34,53]. Similar to other RAB family proteins, RAB11A cycles between a GDP-bound (inactive) and GTP-bound (active) state. When GTP-bound, RAB11A interacts with so-called effector proteins and, in this way, controls the fate and function of the ARE. In vitro experiments revealed that the VPS33B–VIPAR complex, but not VPS33B or VIPAR alone, interacted with GTP-bound RAB11A. The ARC-associated VPS33B-Leu30Pro mutant (Supplemental Table S1) was able to interact with VIPAR but not with RAB11A [30], suggesting that it is the VP333B–VIPAR complex that acts as a RAB11A effector. Other ARC-associated *VPS33B* variants cause a reduced expression of the VPS33B protein, presumably due to the misfolding and degradation of the mutant protein. Depletion of the Vps33b protein in mouse hepatocytes caused elevated serum bile acids, reproducing the cholestasis presented in ARC [65,66]. 

Mechanistically, the loss of the Vps33b protein in mouse hepatocytes caused the mislocalization of the abcb11 protein from the bile canalicular plasma membrane domain to intracellular compartments [65]. In addition, the loss of Vps33b resulted in the mislocalization of other bile canalicular proteins such as Abcb4, Atp8b1 and the cholesterol transporter protein Abcg5/8, but not of the bile canalicular Abcc2 protein or the sinusoidal organic anion transporter protein Slco1b2 [65]. In contrast to other canalicular transporters, Abcc2 does not traffic via Rab11A-positve ARE [67], suggesting that Vps33b specifically regulates ARE-mediated trafficking at the bile canalicular domain. 

Together, these data indicate that the VPS33B–VIPAR protein complex acts as an effector of GTP-bound RAB11A at ARE. Conceivably, *VPS33B* or *VIPAS39* variants result in a loss-of-function of the VPS33B–VIPAR–RAB11A protein complex. This would then inhibit the vesicular trafficking of ABCB11 and other bile canalicular proteins from RAB11A-positive ARE to the bile canalicular domain, and result in the mis-sorting of ABCB11 to the basolateral surface (Figure 3), presumably through impaired membrane fusion events during this journey. Future studies on VPS33B and VIPAR are expected to provide insight into the poorly understood mechanisms via which recycling vesicles fuse with the bile canalicular domain [44,68,69].

### 2.3. MYO5B

**Figure 4:**
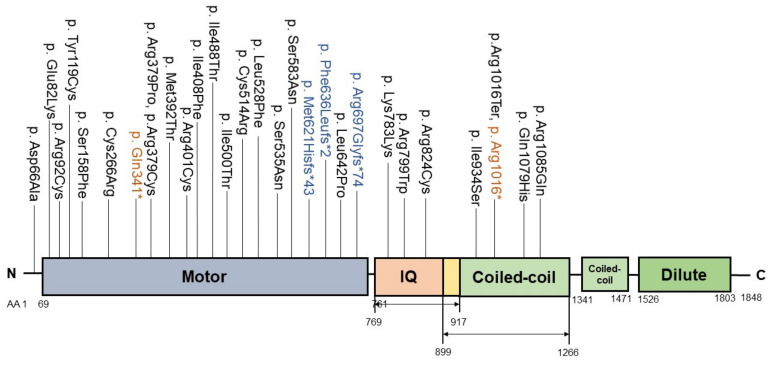
Cholestasis-associated myosin Vb mutations associated with the different myosin Vb protein domains. The protein domains include the motor domain (purple), the isoleucine/glutamine (IQ)-rich domain (orange), the coiled-coil domains (light green) and the Dilute domain (green). N: aminoterminus; C: carboxylterminus; AA: amino acid; *: premature termination.

*MYO5B* variants were identified in patients with intrahepatic cholestasis and low/normal GGT serum levels [24,25] (Supplemental Table S1; Figure 4). *MYO5B* variants can also cause microvillus inclusion disease (MVID) [24], a severe congenital diarrheal and malabsorption disorder requiring life-long total parenteral nutrition [70]. Interestingly, low/normal GGT cholestasis is a frequent symptom of MVID [71]. Whether cholestasis in MVID patients is due to their *MYO5B* variants or is iatrogenic (e.g., due to total parenteral nutrition) is not clear [71]. 

*MYO5B* is located on chromosome 18q21.1 and encodes the actin-based unconventional molecular motor protein myosin Vb. The myosin Vb protein contains an actin filament-binding ATPase motor domain, isoleucine/glutamine (IQ) domains, a coiled-coil region and a cargo-binding carboxy-terminal tail domain, and variants have been found in each domain [24,72] (Figure 4). Through the coiled-coil domains two-headed dimers are formed that move in a stepwise processive manner along actin filaments. Myosin Vb, similar to the VPS33B–VIPAR protein complex, interacts with GTP-bound RAB11A at ARE [73]. 

**Figure 5:**
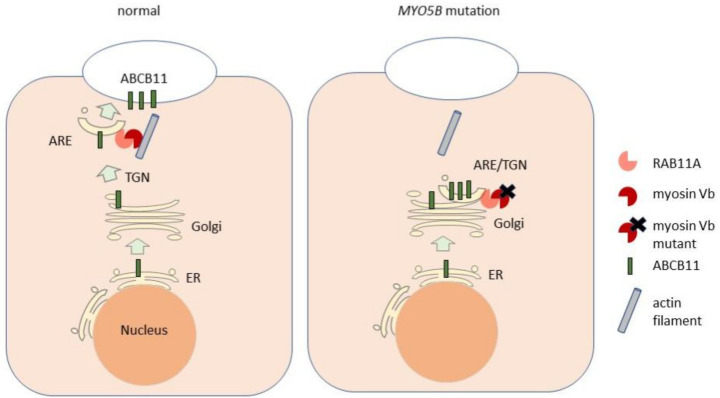
Cartoon depicting the role of myosin Vb and mutant myosin Vb in the trafficking of ABCB11. In the normal situation (left) newly synthesized ABCB11 traffics via the endoplasmic reticulum (ER), the Golgi apparatus and the trans-Golgi network (TGN) to the apical recycling endosomes (ARE). From the ARE, ABCB11 is trafficked to the bile canalicular surface via a mechanism that involves the interaction between the ARE-associated RAB11A and the myosin Vb protein, the latter of which interacts with actin filaments. In the presence of a mutant form of myosin Vb (right), ABCB11 is trapped in a compartment that contains RAB11A, the mutant myosin Vb protein and markers of the TGN, and fails to reach the bile canalicular surface (see also text).

Liver biopsies of patients with intrahepatic cholestasis and *MYO5B* variants revealed a reduced expression and partial mislocalization of ABCB11, and in some cases also of ABCC2 and ABCB4 [24,25,74]. Conceivably, detrimental effects of *MYO5B* variants on bile canalicular protein trafficking mechanistically underlie the intrahepatic cholestasis. Notably, no homozygous *MYO5B* variants that lead to the loss of myosin Vb protein expression (e.g., nonsense, frameshift variants), or to the premature truncated myosin Vb proteins that lack the RAB11A binding sites, have been associated with isolated cholestasis [24,26] (Supplemental Table S1). Moreover, the depletion of myosin Vb from mouse livers, cultured hepatoma cell lines or induced pluripotent stem cell (iPSC)-derived hepatocytes, or the expression of nonsense *MYO5B* mutants in these cells, did not cause the mislocalization of bile canalicular transporter proteins ABCC2 and anoctamin-6 in the steady state [74]. By contrast, the expression of a cholestasis-associated missense myosin Vb mutant (myosin Vb-P660L) or the expression of motorless mutants that expressed only the RAB11A-binding tail domain caused the mislocalization of these bile canalicular transporters [74], in agreement with an earlier report [53]. Furthermore, this effect was shown to require the RAB11A binding site (Y1714) in the carboxy-terminal domain of the mutant myosin Vb, and to require the presence of active, GTP-bound RAB11A [74]. Notably, although the myosin Vb-P660L mutant maintained its colocalization with RAB11A, both were redistributed from the bile canalicular region to the juxtanuclear region, where they colocalized with *trans*-Golgi network marker proteins. Together, these results suggest that the mechanism via which *MYO5B* variants cause cholestasis may not reflect a loss-of-function of myosin Vb, but a gain-of-toxic function of the mutant myosin Vb protein on active RAB11A at the interface of the trans-Golgi network and ARE [74] (Figure 5). 

The mechanism via which myosin Vb mutants inhibit the trafficking of bile canalicular proteins associated with RAB11A-positive ARE or transport vesicles to the bile canalicular membrane remains to be determined. It will be of interest to determine what effect the presence of such myosin Vb mutants may have on the interaction between RAB11A and the VPS33B–VIPAR protein complex.

### 2.4. AP1S1

**Figure 6:**
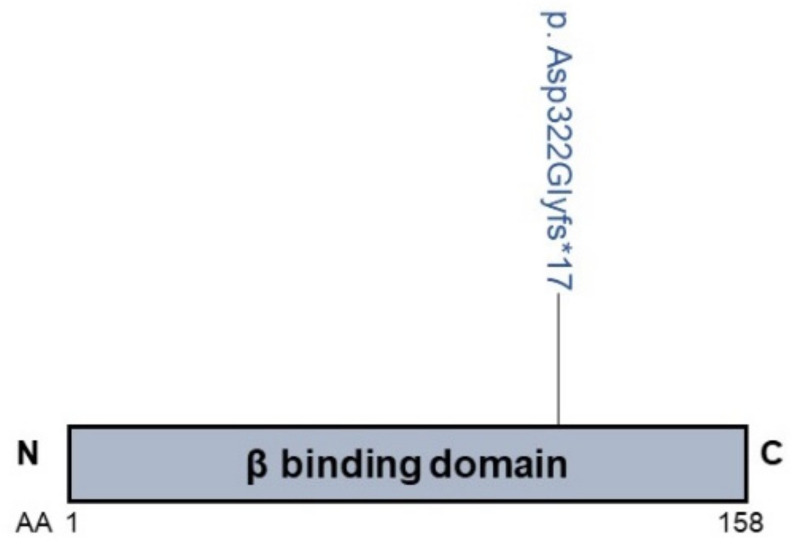
Cholestasis-associated AP1S1 mutation. The beta-binding domain (responsible for interaction with the beta-subunit of the AP1 complex) is shown. N: aminoterminus; C: carboxylterminus; AA: amino acid; *: premature termination.

*AP1S1* variants were identified in patients with MEDNIK (mental retardation, enteropathy, deafness, peripheral neuropathy, ichthyosis, keratodermia) syndrome [32]. Martinelli and colleagues [32] reported that seven MEDNIK patients with a homozygous p.Asp322GlyfsX17 frameshift variant in the *AP1S1* gene presented with intrahepatic cholestasis, as indicated by the elevated total bile acids, the elevated aspartate aminotransferase/alanine aminotransferase ratio, the elevated alkaline phosphatase and transaminases levels, and the normal GGT levels in most cases (Supplemental Table S1; Figure 6). Patients also presented with defects in copper metabolism and liver copper overload [32]. 

*AP1S1* is located on chromosome 7q22.1 and encodes the sigma 1A subunit of the clathrin adaptor protein complex (AP)1. This cytoplasmic AP1S1 protein forms a heterodimer with the gamma subunit in a larger four-subunit complex that also includes an alpha and mu subunit. The AP1 complex localizes both at the *trans*-Golgi network (TGN) and at RAB11A-positive ARE. Here, the AP1 complex is involved in the sorting of proteins that contain dileucine- ([DE]XXXL[LI], where D is aspartate, E is glutamate, L is leucine, I is isoleucine and x is any amino acid) or tyrosine- (YxxΦ, were Y is tyrosine and Φ is a bulky hydrophobic amino acid) based AP1 recognition motifs in their cytoplasmic domains into clathrin-coated transport vesicles [75]. Another AP complex, AP2, localizes at the bile canalicular plasma membrane and recognizes the same motifs.

Causality between *AP1S1* variants and cholestasis has thus far not been demonstrated in cellular or animal model systems. This is necessary as normal liver function tests were reported in another MEDNIK patient [76]. We envision two mechanisms via which *AP1S1* variants may cause cholestasis. Possibly, AP1S1 in the AP1 complex contributes to the sorting and trafficking of ABCB11, which contains a tyrosine-based recognition motif (1306-QKGAY**Y**KL**V**T-1315) and is a known target for the AP2 complex at the canalicular surface [77,78]. Other transporters involved in bile acid homeostasis may also be affected. Indeed, when we analyzed the sequences of proteins associated with bile acid homeostasis-related gene ontologies, we found ten proteins with [DE]XXXL[LI] or YxxΦ motifs in their cytoplasmic domains (Supplemental Table S2). Whether these proteins are true AP1 (and/or AP2) targets needs to be determined experimentally. Alternatively, the loss of AP1S1 could lead to cholestasis via its effect on cellular copper levels. This is supported by the observation that both cholestasis and hepatic copper overload in MEDNIK patients were resolved upon zinc acetate treatment [32]. Elevated copper levels have been shown to impair FXR activity and the transcription of FXR target genes [79], and elevated copper levels in this way possibly reduce the expression of bile acid transporters such as ABCB11. In line with this, loss-of-function variants in the FXR-encoding gene *NR1H4* also cause intrahepatic cholestasis associated with the loss of ABCB11 expression [23]. 

### 2.5. SCYL1

**Figure 7:**
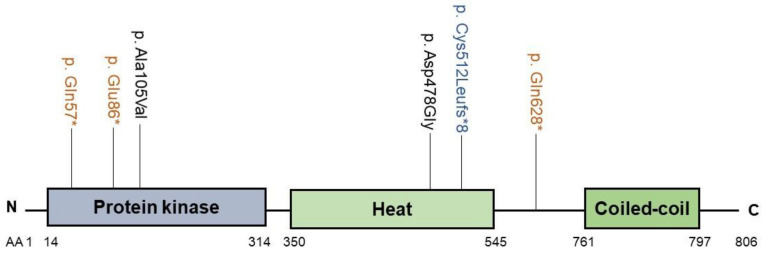
Cholestasis-associated SCYL1 mutations. The protein kinase domain (purple), heat domain (light green) and coiled-coil domain (green) are shown. N: aminoterminus; C: carboxylterminus; AA: amino acid; *: premature termination..

Variants in SCYL1 were identified using exome sequencing in individuals with infantile cholestasis or acute liver failure of unknown etiology [33] (Supplemental Table S1; Figure 7). Patients presented with fever-triggered low GGT recurrent intrahepatic cholestasis episodes during infancy, and variable neurological features. It was suggested to name this disease CALFAN syndrome (low GGT cholestasis, acute liver failure and neurodegeneration) [33]. 

*SCYL1* is located on chromosome 11q13.1 and encodes the Streptomyces cytoskeletal (SCY)1-like protein 1 (SCYL1). SCYL1 is a member of the SCY1-like family of catalytically inactive protein kinases. SCYL1 localizes to the endoplasmic reticulum–Golgi intermediate compartment, and to the *cis*-Golgi, where it recruits components of coatomer-I (COPI) coats. The loss of SCYL1 in non-hepatic cell lines disrupted COPI-mediated retrograde traffic from the Golgi to the endoplasmic reticulum [80]. SCYL1 also localizes to the *trans*-Golgi through the SCYL1-binding protein GORAB [81]. The loss of SCYL1 resulted in the aberrant localization of Golgi-associated enzymes involved in *N*-linked glycosylation [81]. Pathological glycosylation patterns, resembling generalized *N*-glycosylation deficiency, were observed during an episode of liver dysfunction in CALFAN patients’ serum [33]. Loss of SCYL1 has also been shown to affect the morphology of the Golgi, and a fragmented Golgi was observed in liver biopsies of CALFAN patients [33]. The mechanism via which *SCYL1* variants cause cholestasis remains to be determined. As the *N*-linked glycosylation of ABCB11 has been demonstrated to be required for its trafficking to the canalicular plasma membrane [82], it would be of interest to examine the role of SCYL1 in the glycosylation and/or trafficking of bile acid transporters in the secretory pathway. Because the recurrent cholestasis in patients with CALFAN syndrome is triggered by fever episodes, it is possible that the *SCYL1* variants reflect temperature-sensitive mutations that impair SCYL1 protein function at non-permissive higher temperatures.

## 3. Concluding Remarks and Future Perspectives

Multiple newly discovered intrahepatic low/normal GGT cholestasis-associated gene variants appear to associate with intracellular traffic control mechanisms. These underscore intracellular trafficking—and in particular the Golgi- and ARE-mediated trafficking of bile canalicular transporter proteins—as a prominent mechanism to secure normal bile flow in the enterohepatic circulation. 

It thus appears that genes associated with low/normal GGT intrahepatic cholestasis can be categorized into at least two main groups: (i) genes that encode canalicular transporters (e.g., *ABCB11*, *ATP8B1*) and (ii) genes that encode proteins that regulate the trafficking of (some of) these canalicular transporters (Supplemental Table S1). Because genes belonging to this latter group are typically expressed also in other organs, it is not surprising that patients with variants in these genes are more likely to show extra-hepatic manifestations. 

Some cholestasis-associated gene variants that affect intracellular trafficking appear to affect the trafficking and subcellular localization of multiple bile canalicular transporter proteins (e.g., ABCB11, ATP8B1 and/or ABCB4). Therefore, intrahepatic cholestasis caused by these mutated genes can be expected to clinically present with features of multiple classical PFICs/BRICs.

The outstanding questions are how myosin Vb and the VPS33B/VIPAR protein complex—both effectors of RAB11A—function together in time and space with RAB11A at ARE in normal physiology, and, when mutated, in cholestasis. Causality between *AP1S1* variants and cholestasis needs to be addressed in animal or cell studies. MEDNIK patient-specific iPSC-derived bile canaliculi-forming hepatocytes [82] may be instrumental in this. Likewise, CALFAN patient-specific iPSC [83] may be instrumental to address the hepatocellular mechanism underlying *SCYL1* variant-associated cholestasis. 

The enrichment of traffic-regulating genes in idiopathic cases of intrahepatic cholestasis makes it likely that other traffic-regulating genes may also be involved. Therefore, genes that encode regulators of (ARE- or Golgi-mediated) protein trafficking should be considered as potential causative genes when analyzing gene sequencing results from patients with idiopathic intrahepatic cholestasis. Aided by the knowledge of these genes, the further understanding of bile canalicular protein trafficking mechanisms may uncover therapeutic targets to improve bile flow in the enterohepatic circulation. 

## Data Availability

No new data were created or analyzed in this study. Data sharing is not applicable to this article.

## Supplementary Materials

The following are available online at https://www.mdpi.com/2079-7737/10/2/119/s1.

## Abbreviations

ABC	ATP Binding Cassette
ARC	arthrogryposis–renal dysfunction–cholestasis
ASBT	apical sodium bile acid trans
BSEP	bile salt export pump
CALFAN	low gamma-glutamyltransferase cholestasis, acute liver failure and neurodegeneration
COPI	coatomer-I
E17G	estradiol-17beta-d-glucuronide
FIC	familial intrahepatic cholestasis
FXR	farnesoid X receptor
GDP	guanosine diphosphate
GGT	gamma glutamyltransferase
GPI	glycosylphosphatidylinositol
GTP	guanosine triphosphate
MDR	multidrug resistance
MEDNIK	mental retardation, enteropathy, deafness, peripheral neuropathy, ichthyosis, keratodermia) syndrome
MRP	multidrug-resistance protein
MVID	microvillus inclusion disease
NTCP	sodium taurocholate cotransporting polypeptide
OST	organic solute transporter
PFIC	progressive familial intrahepatic cholestasis
SLC	solute carrier
SNARE	soluble N-ethylmaleimide–sensitive factor activating protein receptor
TGN	trans-Golgi network
TUDC	tauroursodeoxycholate
