# Supplementary material for: A Link between Intrahepatic Cholestasis and Genetic Variations in Intracellular Trafficking Regulators
Source: Biology (Basel). 2021 Feb 4;10(2):119. doi: 10.3390/biology10020119 (PMC7914782; doi:10.3390/biology10020119)
Supplement: Supplementary file 1 [file biology-10-00119-s001.zip › biology-1064204-supplementary.pdf]

biology

Table S1. showing proteins involved in bile acid handling with potential AP1/2 dileucine- and/or tyrosine-based recognition motifs. GO: gene ontology. .

Gene/ Protein	Gene alterations	Protein alterations	Status	Protein expression	Biomedical analysis	PMID
VPS33B/ VPS33B	c.707A>T; c.239+5G>A	p.Asp236Val; unknown	Het[1]; Het	n.r.[2]	Increased Tbil[3], normal GGT[4]	31777725
	c.97-2A>C	Unknown	Hom[5]	n.r.	Increased Tbil/Dbil[6] and ALP[7]; normal ALT[8], AST[9] and GGT	29624233
	c.701-(8_5)delTGTT insGA	Unknown	Hom	n.r.	n.r.	24415890
	c.1594C > T	p.Arg532*	Hom	n.r.	Increased Tbil and Dbil; normal GGT	29046204
	c.1157A > C	p.His386Pro	Hom	n.r.	Low GGT	31240160
	c.1246C>T; c.1225+5G>C	p.Arg416*; unknown	Het; Het	n.r.	Increased ALT, AST, ALP and total bile acids; but normal Tbil and GGT	31343487
	c.1225 + 5G>C and partial deletion	Unknown	Unknown	n.r.	Normal GGT and normal Tbil	31463585
	c.1609_1657+9del; c.1225+5G>C	Unknown	Het; Het	n.r.	n.r.	26505894
	c.1225+5G > C; c.440_499del	Unknown; p.Pro147Argfs*4	Het; Het	n.r.	Elevated serum bile acids; normal Tbil and GGT	29907094
	c.700+1G>A; c.1312C>T	Unknown; p.Arg438*	Het; Het	n.r.	Increased Tbil, conjugated bilirubin and ALP; slightly elevated AST; normal ALT and GGT	17994566
	c.403+2T>A; c.239+5G>A	p.Gly134_Asp135ins (GDKR*); p.Gln60Ilefs*22	Het; Het	n.r.	n.r.	21851503; 24917129
	c.403+2T>A; c.499 -11G>A	p.Gly134_Asp135ins (GDKR*); p.Leu166_Glu167ins SerLeuGln	Het; Het	n.r.	Mildly elevated GGT	
	c.239+5G>A; c.621G>A	p.Gln60Ilefs*22; p.Trp207*	Het; Het	n.r.	n.r.	
	c.403+2T>A; c.1509_1510insG	p.Gly134_Asp135ins (GDKR*); p.Lys504Glufs*23	Het; Het	n.r.	Increased Tbil and Dbil; low GGT and normal/slightly increased AST and ALT (Korean patient)	19274792
	c.403+2T>A; c.790_791del	p.Gly134_Asp135ins (GDKR*); p.Tyr267fs*1	Het; Het			
	c.403+2T>A; c.661C>A	p.Gly134_Asp135ins (GDKR*); p.Arg221*	Het; Het			
	c.403+2T>A	p.Gly134_Asp135ins (GDKR*)	Hom			
	c.403+2T>A; unknown	p.Gly134_Asp135ins (GDKR*); unknown	Unkown			
	c.240-577_290-156del; c.1225+5G>C	p.Leu81Serfs*5; unknown	Het; Het	c.1225+5G>C VPS33B mutant retains some ability to interact with VIPAR in vitro	Increased serum bile acids	22753090
	c.1261_1262del; c.1225+5G>C	p.Gln421Valfs*8; unknown	Het; Het		Mild cholestasis	
	c.67C>T	p.Arg23*	n.r.		n.r.	
	c.178-2A>C	Unknown	n.r.		n.r.	
	c.178-1G>C	Unknown	n.r.		n.r.	
	c.711del	p.Phe237Leufs*2	n.r.		n.r.	
	c.853-3C>G	Unknown	n.r.		n.r.	
	c.1030+5G>T	Unknown	n.r.		n.r.	
	c.1498G>T	p.Glu500*	n.r.		n.r.	
	c.240-1G>C	Unknown	n.r.		n.r.	
	c.1657+1G>A	Unknown	n.r.		n.r.	
	c.(?_-354)_(*431+d127_?)del	No predicted protein product	n.r.		n.r.	

	c.1593C>T	p.Arg532*	Hom	n.r.	Normal GGT and normal/slightly elevated ALT and AST	16896922
	c.1311C>T	p.Arg438*	Hom			
	c.89T>C	p.Leu30Pro	Hom			
	c.1518C>T	p.Arg507*	Hom			
	c.319C>T; c.403+1G>T	p.Arg97*; p.Gly134_Asp135ins (GDKR*)	Het; Het			
	c.556_557delCT	p.Leu175fs*219	Hom			
	c.177+1G>A	Unknown	Hom			
	c.853A>G; c.1518C>T	p.Arg507*; unknown	Het; Het			
	c.940–2G>A; ?c.240–13delTT	Unknown	Het; Het			
	c.1406–2A>G	Unknown	Hom			
	c.498+1G>A	Unknown	Het			
	c.1406–1G>C	Unknown	Hom			
	c.348delC	p.Ser116fs*136	Hom			
	c.151C>T; c.433_442delTTGC TGCCTC	p.Arg51*; p.Leu145fs*151	Het; Het			
	c.1208delT	p.Leu403fs*414	Het			
	c.277C>T; c.369_370delTG	p.Arg93*; p.Cys123*	Het; Het			
	c.403+2 T>A; c.1509_1510insG (same as citation 13)	p.Gly134_Asp135ins (GDKR*); p.Lys504Glufs*23	Het; Het	n.r.	Mildly elevated conjugated bilirubin, **elevated GGT**, ALP and total bile acids, but normal ALT and AST (Chinese patient)	24782640
	c.740_741delAT	p.Tyr247*	Het	n.r.	n.r.	18853461
	c.403+2 T>A; c.728C>T	p.Gly134_Asp135ins (GDKR*); p.Ser243Phe	Het; Het			
	c.403+2 T>A; c.1803_1804insA	p.Gly134_Asp135ins (GDKR*); p.Val602Serfs*13	Het; Het			
	c.1235_1236delCCi nsG	p.Pro12Argfs*7	Hom			
	c.1576_1577insT	p.Glu525Valfs*13	Hom			
	c.1594 C>T	p.Arg532*	Het			
	c.352C>T	p.Gln118*	Hom			
	c.853-2 A>G/ c.1519C>T	Unknown; p.Arg507*	Het; Het			
	c.1312C>T	p.Arg438*	Hom			
	c.G700C	p.Asp234His	Hom	n.r.	Almost normal serum GGT level, and markedly elevated ALP level	18347289
	c.1726T>C	p.Cys576Arg	Hom	VPS33B$^{c.1726T>C, p.Cys576Arg}$ caused decreased protein expression and abolished interaction with VIPAS39 in-vitro	Mild cholestasis, with normal GGT	31479177
	c.1726T>C; c. 1509dupG	p.Cys576Arg; p.(Lys504GlufsTer)	Het; Het		Mild elevated total bile acids; normal bilirubin, GGT	
	c.1033delA; c.1567C>T	p.Ile345Leufs*8; p.Arg523*	Het; Het	n.r.	Markedly elevated Tbil, Dbil and ALP; mild elevation of ALT and AST; normal GGT	24415890
	IVS6+1G>A	Unknown	Hom			15052268
	c.1518C>T; IVS13-2A>G	p.Arg507*; unknown	Het; Het			
	c.971delA	p.Lys324fs	Hom	n.r.	Increased Tbil, Dbil, ALT and ALP; normal GGT	16492441
***VIPAS39*/ VIPAR**	c.1090G>A	p.ILe364Leu	Het	n.r.	n.r.	24917129
	c.638T>C	p.Leu213Pro	n.r.	n.r.	n.r.	22753090
	c.463_464del	p.Trp155Glufs*4	n.r.			
	c.484C>T	p.Arg162*	n.r.			
	c.837-1G>T	Unknown	n.r.			
	c.177_179delinsAA	p.Trp59Ter;	Het; Het	n.r.	Normal GGT	31479177

	A; c.1141C>T	p.Arg381Ter				
	c.373A>T	p.Arg125*	Hom	n.r.	Normal GGT; High bile acids	28039895
	c.1130G>C	p.Arg377Pro	Hom	n.r.	Greatly increased ALP; normal GGT	26808426
	c.1021T>C	p.Cys341Arg	Hom	No mutation was found in VPS33B	Normal GGT, increased serum bile acid concentration	23636179
***MYO5B*/ Myosin Vb**	c.1540T>C	p.Cys514Arg	Hom	n.r.	Normal GGT; High bile acids	28039895
	c.3254G > A	p.Arg1085Gln	Het	n.r.	n.r.	29707407
	c.197A > C	p.Asp66Ala	Het			
	c.3538-1G>A; c.241415G>T	Splicing; Splicing	Het; Het	n.r.	Elevated Dbil and total bile acid concentration; mildly elevated ALT and AST; low GGT	28027573
	c.1201C>T; c.1021C>T	p.Arg401Cys; p.Gln341*	Het; Het			
	c.3237G>C; c.1604G>A	p.Gln1079His; p.Ser535Asn	Het; Het			
	c.796T>C	p.Cys266Arg	Hom			
	c.1748G>A; c.2801T>G	p.Ser583Asn; p.Ile934Ser	Het; Het			
	c.2090_2090delG; c.4852111A>G	p.Arg697Glyfs*74; Splicing	Het; Het			
	c.3046C>T; c.437C>T	p.Arg1016*; p.Ser158Phe	Het; Het			
	c.2470C>T	p.Arg824Cys	Hom			
	c.2470C>T	p.Arg824Cys	Het		n.r.	
	c.1136G>C	p.Arg379Pro	Het		n.r.	
	c.274C>T; c.2395C>T	p.Arg92Cys; p.Arg799Trp	Het; Het	n.r.	Slightly elevated AST; normal ALT and GGT	27532546
	c.1499T>C; c.1925T>C	p.Ile500Thr; p.Leu642Pro	Likely to be Het; Het		Elevated ALT and AST; normal GGT	
	c.356A>G	p.Tyr119Cys	Hom		Elevated ALT and AST; normal GGT	
	c.1135C>T; c.1906-2A>G; c.2470C>T	p.Arg379Cys; splicing, Phe636Leufs*2; p.Arg824Cys	Het; Het		Mildly elevated ALT and AST; normal GGT	
	c.1753-1G>T; c.2395C>T	Splicing; p.Arg799Trp	Het; Het		Mildly elevated ALT and AST; normal GGT	
	c.1222A>T; c.1582C>T	p.Ile408Phe; p.Leu528Phe	n.r.	n.r.	Low GGT	25111220
	c.274C>T; c.1463T>C	p.Arg92Cys; p.Ile488Thr	Het; Het	n.r.	High Tbil, ALT and serum bile acids; normal GGT	32304554
	c.1860dupT; c.274C>T	p.Met621Hisfs*43; p.Arg92Cys	Het; Het		High serum bile acids; normal Tbil, ALT	
	c.2470C>T	p.Arg824Cys	Hom		High serum bile acids;normal Tbil, ALT, GGT	
	c.1175T>C; c.2349A>G	p.Met392Thr; p.Lys783Lys	Het; Het		High Tbil, ALT and serum bile acids; normal GGT	
	c.3046C>T; c.1175T>C	p.Arg1016Ter; p.Met392Thr	Het; Het		High Tbil, ALT and serum bile acids; normal GGT	
	c.244G>A	p.Glu82Lys	Hom		High Tbil, ALT and serum bile acids; normal GGT	
***AP1S1*/ AP1S1**	IVS2-2A>G	Unknown	n.r.	n.r.	Raised total bile acids, ALP and AST/ALT; normal GGT	23423674
	c.356_365insG	p.Asp322Glyfs*17	Hom	Complete absence of AP1S1 protein		
***SCYL1*/ SCYL1**	c. 1882C > T	p.Gln628*	Hom	Expression of SCYL1 was severely reduced	Increased AST and ALT, marginally elevated GGT	29419818
	c. 1433A > G	p.Asp478Gly	Hom	Expression of SCYL1 was severely reduced		
	c. 256G > T	p.Glu86*	Hom	Unknown		
	c. 169C > T	p.Gln57*	Hom	Unknown		
	c. 314C > T	p.Ala105Val	Hom	Expression of SCYL1 was severely reduced		
	c.1534dupT	p.Cys512Leufs*8	Hom	A true protein truncation	n.r.	30531813

or nonsense-mediated messenger RNA decay

Abbreviations:
fs frameshift, del deletion, ins insertion, * stop, IVS intervening sequence, dup duplication
Het[1] Heterozygous,
n.r.[2] Not reported,
Tbil[3] total bilirubin
GGT[4] gamma glutamyl transferase,
Hom[5] Homozygous,
Dbil[6] direct bilirubin
ALP[7] alkaline phosphatase,
ALT[8] alanine aminotransferase,
AST[9] aspartate aminotransferase

Supplemental Table S2

GO Term	Protein name	Motif (D/ExxxL[L/I]; Yxxphi)
0032782	Phosphatidylcholine translocator ABCB4	1011-F**E**RQP**LI**DSY-1020;<br>361-RGAA**Y**VI**F**DI-370;<br>511-A**Y**EF**I**MKLPQ-520;<br>621-EGV**Y**FK**L**VNM-630;<br>921-EKL**Y**GP**Y**RNS-930;<br>1081-LLERF**Y**DP**L**A-1090
0015721	Bile salt export pump	536-NA**Y**NF**I**MDLP-545;<br>646-RKGV**Y**FT**L**VT-655;<br>1306-QKGA**Y**YK**L**VT-1315;<br>1306-QKGAY**Y**KL**V**T-1315;
0015721	Canalicular multispecific organic anion transporter 1	531-K**E**LKN**LL**AFS-540;<br>731-QQVL**E**ACA**LL**-740;<br>881-ED**D**DYG**LI**SS-890;<br>1466-AVDL**E**TDN**LI**-1475;<br>201-LSSIT**Y**SW**Y**D-210;<br>726-NEKR**Y**QQ**V**LE-735;<br>771-LARAT**Y**QN**L**D-780;<br>841-EKGS**Y**SA**L**LA-850;<br>881-EDDD**Y**GL**I**SS-890;<br>961-KVKFSI**Y**LE**Y**-970;<br>1156-RSPI**Y**SH**F**SE-1165;<br>1271-RITE**Y**TK**V**EN-1280
0015721	Canalicular multispecific organic anion transporter 2	281- SG**E**DEV**LL**GA-290;<br>721- QQTL**E**ACA**LL**-730;<br>871- AE**D**KEA**LL**IE-880;<br>1456- IDL**E**TDN**LI**Q-1465;<br>61- RG**Y**II**L**SHLS-70<br>121- LLIQ**Y**ER**L**QG-130;<br>371- **Y**HY**I**FVTGVK-380;<br>371- YH**Y**IF**V**TGVK-380;<br>831- EMGP**Y**PA**L**LQ-840;<br>1146- PI**Y**SH**F**SETV-1155;<br>1181- NQRSC**Y**PY**I**I-1190;<br>1486- TIMD**Y**TR**V**LV-1495;

0015721	Aquaporin-8	26- WRVSW**Y**ER**F**V-35
0015721	Ileal sodium/bile acid cotransporter	146- LI**Y**TK**M**WVDS-155;<br>306- GF**Y**VA**Y**KKCH-315
0015721	Sodium/bile acid cotransporter 4	381- I**Y**KM**Y**GSEML-390;<br>401- DTDIS**Y**KK**L**K-410;<br>416- DTS**Y**GT**V**KAE-425
0015721	Solute carrier family 10 member 6	181- **Y**VN**Y**RWPKQS-190
0015721	Solute carrier organic anion transporter family member 2B1	221- SNSPL**Y**LG**I**L-230
0038183	G-protein coupled bile acid receptor 1	111- **Y**MA**V**LRPLQP-120
